# Comparison of NMR and crystal structures for the proteins TM1112 and TM1367

**DOI:** 10.1107/S1744309110020956

**Published:** 2010-08-07

**Authors:** Biswaranjan Mohanty, Pedro Serrano, Bill Pedrini, Kristaps Jaudzems, Michael Geralt, Reto Horst, Torsten Herrmann, Marc-André Elsliger, Ian A. Wilson, Kurt Wüthrich

**Affiliations:** a Department of Molecular Biology, The Scripps Research Institute, La Jolla, CA 92037, USA; b Joint Center for Structural Genomics, http://www.jcsg.org, USA; c Institute of Molecular Biology and Biophysics, ETH Zürich, CH-8093 Zürich, Switzerland; d Centre Européen de RMN à Très Hauts Champs, Université de Lyon, F-69100 Villeurbanne, France; e Skaggs Institute of Chemical Biology, The Scripps Research Institute, La Jolla, CA 92037, USA

**Keywords:** NMR and crystal structure comparison, structure-determination software, reference structures, *Thermotoga maritima*

## Abstract

The NMR structures of the TM1112 and TM1367 proteins from *Thermotoga maritima* in solution at 298 K were determined following a new protocol which uses the software package *UNIO* for extensive automation. The results obtained with this novel procedure were evaluated by comparison with the crystal structures solved by the JCSG at 100 K to 1.83 and 1.90 Å resolution, respectively. In addition, the TM1112 solution structure was compared with an NMR structure solved by the NESG using a conventional largely interactive methodology. For both proteins, the newly determined NMR structure could be superimposed with the crystal structure with r.m.s.d. values of <1.0 Å for the backbone heavy atoms, which provided a starting platform to investigate local structure variations, which may arise from either the methods used or from the different chemical environments in solution and in the crystal. Thereby, these comparative studies were further explored with the use of reference NMR and crystal structures, which were computed using the NMR software with input of upper-limit distance constraints derived from the molecular models that represent the results of structure determination by NMR and by X-ray diffraction, respectively. The results thus obtained show that NMR structure calculations with the new automated *UNIO* software used by the JCSG compare favorably with those from a more labor-intensive and time-intensive interactive procedure. An intriguing observation is that the ‘bundles’ of two TM1112 or three TM1367 molecules in the asymmetric unit of the crystal structures mimic the behavior of the bundles of 20 conformers used to represent the NMR solution structures when comparing global r.m.s.d. values calculated either for the polypeptide backbone, the core residues with solvent accessibility below 15% or all heavy atoms.

## Abstract 2

NMR structures of the proteins TM1112 and TM1367 solved by the JCSG in solution at 298 K could be superimposed with the corresponding crystal structures at 100 K with r.m.s.d. values of <1.0 Å for the backbone heavy atoms. For both proteins the structural differences between multiple molecules in the asymmetric unit of the crystals correlated with structural variations within the bundles of conformers used to represent the NMR solution structures. A recently introduced JCSG NMR structure-determination protocol, which makes use of the software package *UNIO* for extensive automation, was further evaluated by comparison of the TM1112 structure obtained using these automated methods with another NMR structure that was independently solved in another PSI center, where a largely interactive approach was applied.

## 1. Introduction   

Crystal structures of *Thermotoga maritima* proteins TM1112 and TM1367 have previously been determined by the JCSG (McMullan *et al.*, 2004 ▶; Jin *et al.*, 2006 ▶). These two proteins were therefore used in an NMR methods development and assessment project to evaluate the NMR structures that were obtained following a novel protocol, which uses the *UNIO* software suite (Herrmann *et al.*, 2002*a* ▶,*b* ▶; Volk *et al.*, 2008 ▶; Fiorito *et al.*, 2008 ▶; Herrmann *et al.*, unpublished work) that supports extensive automation. These structures were incorporated into the present series of NMR and crystal structure comparisons because the crystal structures include two and three independent molecules in the asymmetric unit, respectively, and it seemed of interest to follow up on earlier investigations of possible correlations between variations among the individual molecules in the asymmetric unit of the crystal and the bundle of NMR conformers or between the crystal and NMR structures (see, for example, Wilson & Brunger, 2000 ▶; DePristo *et al.*, 2004 ▶; Furnham *et al.*, 2006 ▶; Levin *et al.*, 2007 ▶; Kondrashov *et al.*, 2008 ▶).

The NMR structure of TM1112, a protein of unknown function (DUF861; PF05899), was initially determined by the Northeast Structural Genomics (NESG) consortium (PDB code 1lkn) and was subsequently used by the JCSG to solve the crystal structure by molecular replacement (http://www.topsan.org/Proteins/JCSG/1o5u). Thus, we include the NESG structure in the following comparative studies. TM1367 also represents a domain of unknown function (DUF369; PF0412; http://www.topsan.org/Proteins/JCSG/2ka0). Its crystal structure (PDB code 1zx8) revealed an atypical cyclophilin-type (peptidylprolyl isomerase-type) fold (Jin *et al.*, 2006 ▶).

Furthermore, to support the comparative studies, we continued to explore the use of reference NMR and crystal structures (Jaudzems *et al.*, 2010 ▶), which were computed from sets of distance restraints measured in the crystal and solution NMR molecular models, respectively, using the same simulated-annealing protocol as for the experimental NMR structure determination.

## 2. Materials and methods   

### 2.1. Preparation of TM1112   

The plasmid vector MH1 containing the TM1112 gene obtained from the JCSG Crystallomics Core was used as the template for PCR amplification with the primers 5′-CCG**CAT*ATG***GAAGTGAAGATAGAAAAGCCCACACCC-3′ and 5′-CGG**AAGCTT** *CTA*GAAGAGGTTGTAGTGCTTTCTGACCGGCTCTAAAAC-3′, where the *Nde*I and *Hin*dIII restriction sites are shown in bold and the initiation and stop codons are italicized. The PCR product was digested with *Nde*I and *Hin*dIII and inserted into the vector pET-25b between the same restriction sites after treatment with calf intestinal alkaline phosphatase (CIP). The resulting plasmid pET-25b-TM1112 was used to transform *Escherichia coli* strain Rosetta (DE3) (Novagen) and the protein was expressed in M9 minimal medium containing 1 g l^−1^ ^15^NH_4_Cl and 4 g l^−1^ [^13^C_6_]-d-glucose (Cambridge Isotope Laboratories) as the sole sources of nitrogen and carbon. After the addition of 100 mg l^−1^ ampicillin, the cells were grown at 310 K to an OD_600_ of 0.45, induced with 1 m*M* isopropyl β-d-1-thiogalactopyranoside (IPTG) and grown for a further 3 h to a final OD_600_ of 1.10. The cells were harvested at 5000*g* and 277 K for 5 min and frozen at 253 K overnight. The next day, the cell pellet was thawed and resuspended in 30 ml buffer *A* (25 m*M* sodium phosphate pH 7.6, 25 m*M* NaCl, 2 m*M* DTT) containing one Complete EDTA-free protease-inhibitor cocktail tablet (Roche) and lysed by ultrasonication. The soluble fraction of the cell lysate was isolated by centrifugation for 30 min at 20 000*g* and 277 K, decanting and filtration through a 0.22 µm pore-size filter. The solution was then incubated in a water bath at 348 K for 20 min. Precipitated material was removed by centrifugation at 6000*g* for 20 min at 277 K. The supernatant was recovered and passed through the aforementioned filter before application onto a 5 ml HiTrap QHP column (GE Healthcare) pre-equilibrated in buffer *A*. TM1112 eluted in the flowthrough during sample injection. These fractions were pooled and concentrated to 12 ml by ultrafiltration using an Amicon ultracentrifugal filter device with 5 kDa molecular-weight cutoff (Millipore) and then applied onto a HiLoad 26/60 column of Superdex 75 gel-filtration resin (GE Healthcare) pre-equilibrated in NMR buffer *A* (25 m*M* sodium phosphate pH 6.8, 50 m*M* NaCl, 0.5 m*M* DTT). The fractions containing TM1112 were pooled and concentrated from 50 ml to 500 µl by ultrafiltration. All purification steps were monitored by SDS–PAGE. The yield of purified TM1112 was 30 mg per litre of culture.

NMR samples were prepared by adding 10%(*v*/*v*) D_2_O, 4.5 m*M* d_10_-DTT and 0.03%(*w*/*v*) NaN_3_ to 500 µl of a 1.3 m*M* solution of ^15^N,^13^C-labeled TM1112 in NMR buffer *A*.

### 2.2. Preparation of TM1367   

The plasmid vector MH4a containing the TM1367 gene obtained from the JCSG Crystallomics Core was used as the template for PCR amplification with the primers 5′-CCG**CAT*ATG***AGAGTTGAACTCCTCTTTGAAAGTGGAAAATGTG-3′ and 5′-CGG**AAGCTT** *CTA*TGAGGATGCAAATCTGACGGCG-3′, where the *Nde*I and *Hin*dIII restriction sites are shown in bold and the initiation and stop codons are italicized. The expression and purification of this protein then followed the same protocol as described in §[Sec sec2.1]2.1 for TM1112, with the following modifications. The cells were resuspended in 24 ml buffer *B* (25 m*M* sodium phosphate pH 6.8, 25 m*M* NaCl, 1 m*M* DTT) containing half of a Complete EDTA-free protease-inhibitor cocktail tablet (Roche). Buffer *B* was also used to pre-equilibrate the 5 ml HiTrap QHP column (GE Healthcare) used in a subsequent purification step, which yielded TM1367 fractions that were pooled into two volumes of 12 ml and applied onto a HiLoad 26/60 column of Superdex 75 gel-filtration resin (GE Healthcare) pre-equilibrated in NMR buffer *B* (25 m*M* sodium phosphate pH 6.0, 50 m*M* NaCl, 0.5 m*M* DTT). The fractions containing TM1367 were again pooled and concentrated from 60 ml to 500 µl by ultrafiltration using an Amicon ultracentrifugal filter device with 5 kDa molecular-weight cutoff (Millipore). The yield of purified TM1367 was 32 mg per litre of culture.

NMR samples were prepared by adding 10%(*v*/*v*) D_2_O, 4.5 m*M* d_10_-DTT and 0.03%(*w*/*v*) NaN_3_ to 500 µl of a 1.3 m*M* solution of ^15^N,^13^C-labeled TM1367 in NMR buffer *B*.

### 2.3. NMR spectroscopy   

NMR experiments were conducted at 298 K on Bruker Avance 600 and 800 MHz spectrometers equipped with TXI HCN *z*-gradient and *xyz*-gradient room-temperature probes, respectively. 4D APSY-HACANH, 5D APSY-HACACONH and 5D APSY-CBCACONH data sets were recorded with 16, 16 and 16 projections, respectively (Hiller *et al.*, 2005 ▶, 2008 ▶). Three NOESY spectra were recorded with a mixing time of 60 ms: 3D [^1^H,^1^H]-NOESY-^15^N-HSQC, 3D [^1^H,^1^H]-NOESY-^13^C(ali)-HSQC and 3D [^1^H,^1^H]-NOESY-^13^C(aro)-HSQC. The ^13^C carrier frequencies were set at 25 and 122 p.p.m., respectively, for obtaining the aliphatic and aromatic spectral regions. Chemical shifts were referenced internally to the 2,2-dimethyl-2-silapentane-5-­sulfonate (DSS) signal (Wishart & Sykes, 1994 ▶). The chemical shift of the solvent water resonance relative to DSS was 4.796 p.p.m.

### 2.4. NMR structure determination   

The polypeptide-backbone resonance assignments were obtained from APSY-generated four- and five-dimensional peak lists, which were used as input for automated backbone assignment with v.2.2 of the program *MATCH* (Volk *et al.*, 2008 ▶) in the *UNIO* software package. The backbone assignments were then interactively checked and completed. Side-chain resonance assignments were obtained with the automated routine of v.2.2 of the program *ATNOS*/*ASCAN* (Herrmann *et al.*, 2002*a* ▶; Fiorito *et al.*, 2008 ▶) in the *UNIO* software package, using as input the aforementioned 3D ^15^N-resolved and ^13^C-­resolved [^1^H,^1^H]-NOESY spectra. The automatic assignments were interactively checked and extended using the software *CARA* (Keller, 2004 ▶). NOE distance restraints were automatically collected using the same three NOESY data sets as for the side-chain assignments as input for v.2.2 of the *ATNOS*/*CANDID* programs (Herrmann *et al.*, 2002*a* ▶,*b* ▶) in the *UNIO* software package. The structure calculation and energy refinement were performed as described in Jaudzems *et al.* (2010 ▶).

### 2.5. Structure validation and data deposition   

Structure validation was performed as described in Jaudzems *et al.* (2010 ▶). The chemical shifts have been deposited in the BioMag­ResBank (entry Nos. 16006 and 16007 for TM1112 and TM1367, respectively; http://www.bmrb.wisc.edu). The atomic coordinates of the bundles of 20 NMR conformers have been deposited in the PDB (accession codes 2k9z for TM1112 and 2ka0 for TM1367; http://www.rcsb.org/pdb/).

### 2.6. Calculation of reference crystal structures and reference NMR structures   

We follow the strategy introduced in Jaudzems *et al.* (2010 ▶). To compute the reference crystal structure, the positions of the H atoms in the crystal were calculated using the standard residue geometry from the AMBER94 library in the software *MOLMOL* (Koradi *et al.*, 1996 ▶). All intra- and inter-residual distances shorter than 5.0 Å between pairs of H atoms were then extracted and those involving labile protons with fast chemical exchange (Wüthrich, 1986 ▶) were eliminated from the resulting list. The input of upper-limit distance bounds for the structure calculation was generated by increasing these proton–proton distances by 15%. This ‘loosening’ of the distance constraints is in line with the basic strategy of interpreting ^1^H–^1^H NOEs in terms of upper-limit distance bounds (Wüthrich, 1986 ▶). For the NMR reference structure, we followed a three-step protocol: (i) a list was prepared of all the ^1^H–^1^H distances shorter than 5.0 Å in the 20 conformers that represent the NMR structure; (ii) a new list was obtained that included the longest distance among the 20 conformers for each pair of H atoms in the list resulting from (i); and (iii) the input of upper-limit distance bounds contained all entries in list (ii) that were shorter than 5.75 Å [this value was empirically selected as the shortest cutoff that gave virtually identical results for the structure calculation as an input consisting of the complete list (ii)].

### 2.7. Calculation of global displacements, global r.m.s.d.s, solvent accessibility and occluded surface packing (OSP)   

The techniques used here have been described in Jaudzems *et al.* (2010 ▶). The global per-residue displacements between structure bundles refer to the mean structures calculated after superposition for minimal r.m.s.d. of the backbone atoms of residues 2–89 for TM1112 and 2–123 for TM1367.

## 3. Results and discussion   

**Figure 1:**
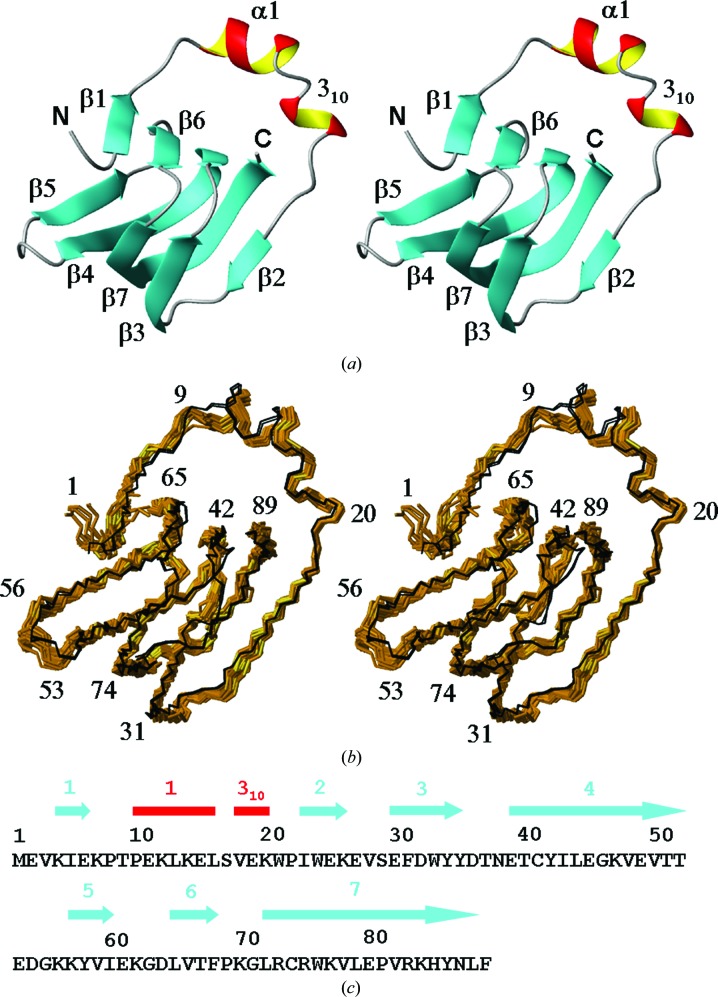
Amino-acid sequence and NMR structure of the protein TM1112 and comparison of the NMR structure with the crystal structure. (*a*) Stereo ribbon diagram of the NMR conformer closest to the mean coordinates of the bundle of conformers in (*b*). Color code: β-strands, cyan; helices, red/yellow; nonregular secondary structure, gray. The individual regular secondary structures are identified and the two chain ends are marked N and C. (*b*) Stereoview of a superposition for best fit of the polypeptide-backbone heavy atoms of residues 2–89 of the two molecules in the crystal asymmetric unit, Cryst*A* and Cryst*B* (black lines), with the bundle of 20 conformers that represent the NMR structure (brown). In generating this picture, Cryst*B* was superimposed for best fit with Cryst*A* and then each one in the ensemble of 20 NMR conformers was superimposed for best fit of the polypeptide-backbone heavy atoms with Cryst*A*. (*c*) Amino-acid sequence. The locations of regular secondary structure are indicated above the sequence using the same color code as in (*a*).

**Figure 2:**
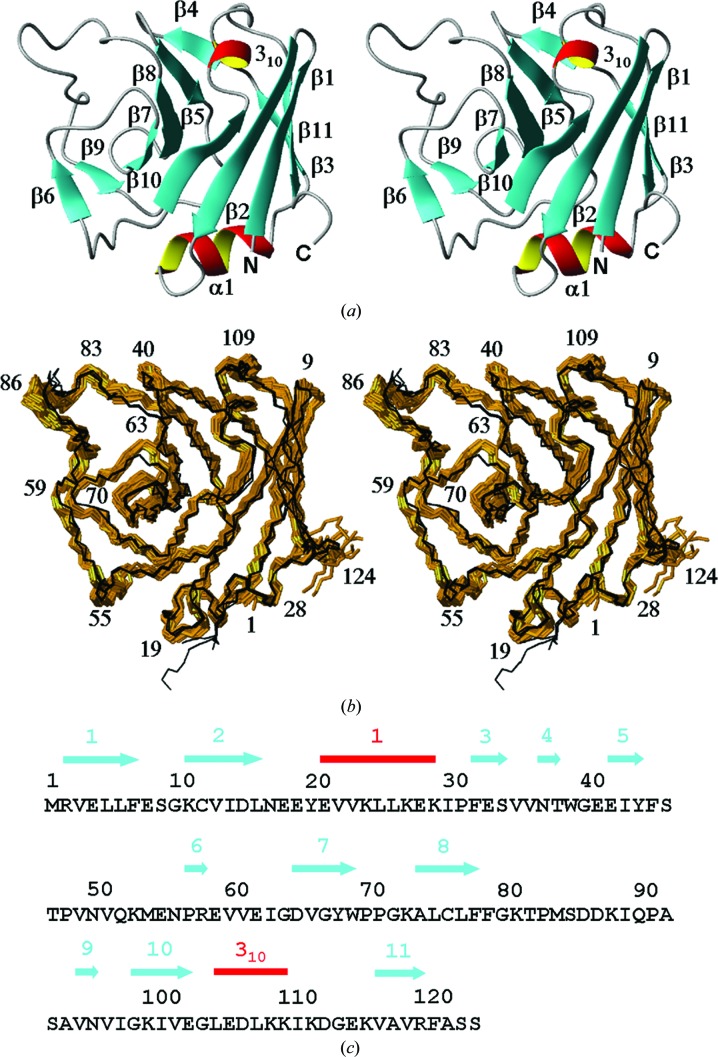
Amino-acid sequence and NMR structure of the protein TM1367 and comparison of the NMR structure with the crystal structure. The same presentation is used as in Fig. 1 ▶.

**Figure 3:**
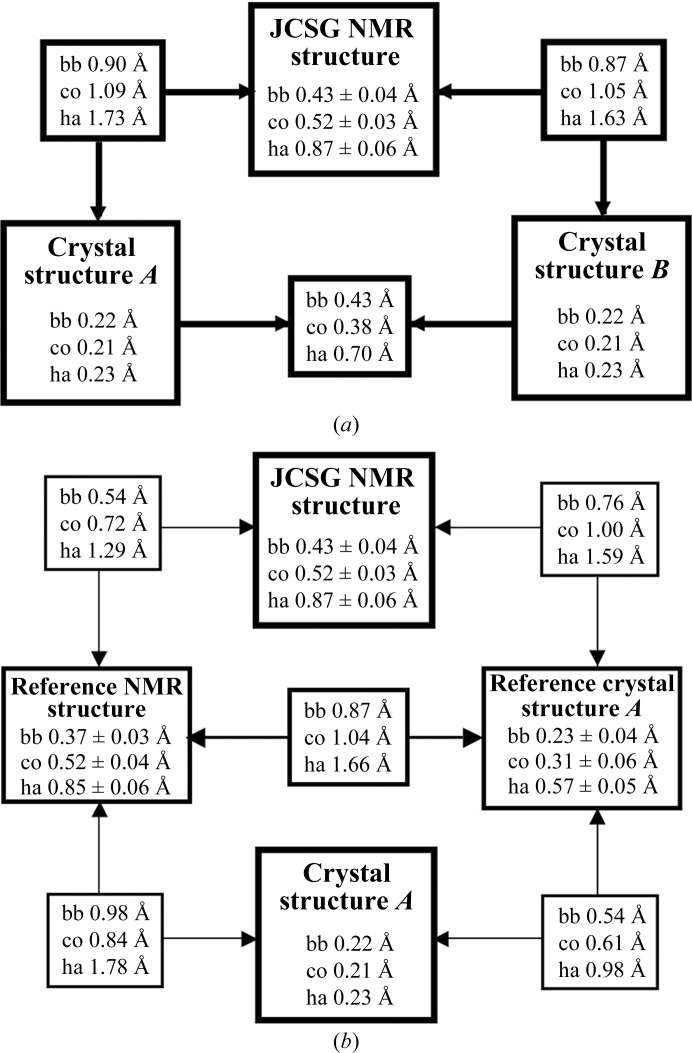
Analysis of the crystal structure, the NMR structure and the reference crystal and NMR structures of TM1112. (*a*) R.m.s.d. values describing the precision of the structure determinations by NMR in solution at 298 K and by X-ray diffraction in crystals at 100 K. The smaller boxes show the r.m.s.d. values for pairwise comparisons between the bundle of 20 NMR conformers and Cryst*A* and Cryst*B*. For the crystal structure, ‘global deviations’ corresponding to r.m.s.d.s were computed from the experimental *B* values using equations (2)–(5) in Jaudzems *et al.* (2010 ▶). For the structure comparisons, r.m.s.d. values for residues 2–89 were computed between the atom coordinates of the indicated crystal structure molecule and those of the conformer closest to the mean atom coordinates of the ensemble of 20 NMR conformers. The atoms used for the comparisons are bb, the backbone atoms N, C^α^ and C′; co, core heavy atoms defined as having less than 15% solvent accessibility; ha, all heavy atoms. (*b*) Corresponding data as in (*a*) for the reference NMR structure, the reference crystal structure computed from input collected with Cryst*A* and for pairwise comparisons with the experimental structures. Numbers framed by thick lines represent the precision of the experimental NMR and crystal structures and their comparisons, those framed by medium lines represent the precision of the reference NMR and reference crystal structures and their comparison and those framed by thin lines represent the comparisons between experimental and reference structures.

**Figure 4:**
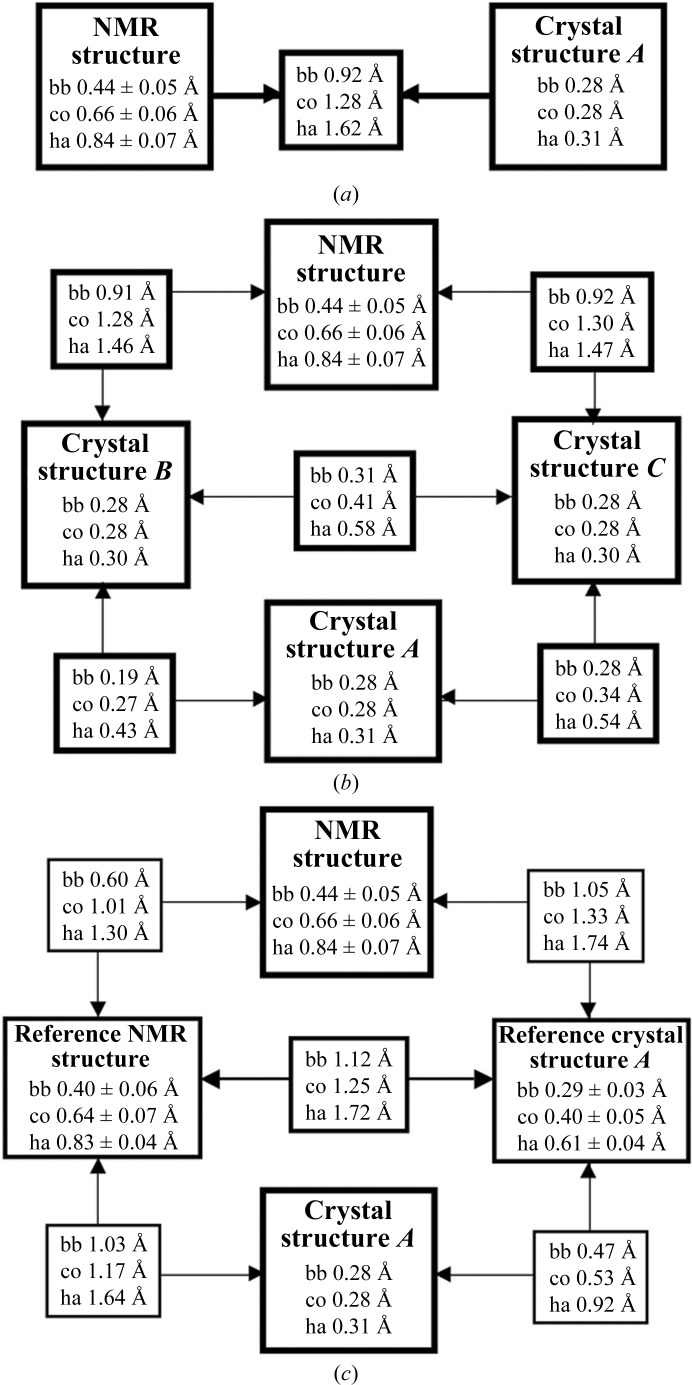
Analysis of the crystal structure, the NMR structure and the reference crystal and NMR structures of TM1367. The same presentation is used as in Fig. 3 ▶.

Comparison of the bundle of 20 conformers representing the NMR structure of TM1112 with the two molecules in the asymmetric unit (a.s.u.) of the crystal structure (denoted here as Cryst*A* and Cryst*B* following the corresponding chain designation; PDB code 1o5u) reveals remarkable similarity (Fig. 1 ▶) and comparable results were obtained for the comparison between the bundle of 20 NMR conformers of TM1367 with the three molecules in the a.s.u. of the crystal structure (denoted here as Cryst*A*, Cryst*B* and Cryst*C* following the corresponding chain designation; PDB code 1zx8; Fig. 2 ▶). For TM1112, the NMR and crystal structures superimpose with global backbone heavy-atom r.m.s.d. values below 1.0 Å for residues 2–89 (Fig. 3 ▶). For TM1367, global r.m.s.d values below 1.0 Å were calculated for the backbone heavy atoms of residues 2–123 (Fig. 4 ▶).

With this starting platform, we aimed here to evaluate the accuracy and precision of the independently determined NMR and crystal structures and to investigate differences that may be associated with the different chemical environments in solution and in the crystal. To this end, we continued to explore a recently introduced approach to reduce possible bias from the different software used for structure determination by the two techniques (Jaudzems *et al.*, 2010 ▶), which is based on the use of reference NMR and crystal structures computed with the NMR software from distance constraints measured in the NMR and crystal structure models, respectively. We now describe the results of these investigations using two *T. maritima* proteins, TM1112 and TM1367, which also include comparisons with an NMR structure of TM1112 solved by the NESG (PDB code 1lkn).

### 3.1. Global comparison of the NMR and crystal structures of TM1112   

The TM1112 NMR structure was solved by the JCSG at 298 K in 20 m*M* sodium phosphate buffer pH 6.8, 50 m*M* sodium chloride, 5 m*M* DTT and 0.03%(*w*/*v*) sodium azide. The JCSG crystal structure was determined at 1.83 Å resolution at 100 K using a crystal obtained at 277 K from 20 m*M* Tris buffer pH 7.9 and 25.5%(*w*/*v*) PEG 4000 (McMullan *et al.*, 2004 ▶). TM1112 comprises seven antiparallel β-­strands (residues 4–6, 23–26, 30–35, 39–52, 57–60, 65–68 and 72–­88), an α-helix (10–16) and a 3_10_-helix (18–20). These regular secondary-structure elements are arranged in the sequential order β1–α1–3_10_–β2–β3–β4–β5–β6–β7 (Fig. 1 ▶). The β-barrel consists of two connecting sheets, β1–β6–β4–β7–β2 and β5–β4–β7–β3, which are coupled together by two highly twisted strands β4 and β7 that extend from one sheet to the other. The helical segment (residues 10–20) is inserted between β1 and β2 on opposite ends of the β-barrel *via* two well defined loops (residues 7–9 and 21–24) and traverses one face of the β-barrel. Statistics for the NMR structure determination are given in Table 1 ▶ and those for the crystal structure have been presented elsewhere (McMullan *et al.*, 2004 ▶).

The reference NMR and crystal structures were calculated from a significantly larger number of upper-limit distance constraints than the experimental NMR structure. The main factors causing the numbers of constraints to be different (Table 1 ▶) are that owing to the limited resolution and sensitivity of the NMR measurements only a fraction of the short ^1^H–^1^H distances are collected in the experimental structure determination, whereas in the aforementioned molecular models all of the short contacts are evaluated. Furthermore, in the present reference structure calculations only the methyl groups were represented by pseudo-atoms (Wüthrich *et al.*, 1983 ▶), whereas in the experimentally collected input the methylene groups and the pairs of symmetry-related ring protons of Phe and Tyr were also represented by pseudo-atoms.

Comparison of the two molecules in the crystal structure with the NMR conformer closest to the mean coordinates of the bundle of 20 NMR conformers (Fig. 3 ▶ *a*) yielded backbone r.m.s.d. values of 0.90 and 0.87 Å and all-heavy-atom r.m.s.d.s of 1.73 and 1.63 Å. The crystal structure and the reference crystal structure exhibit closely similar r.m.s.d.s relative to the experimental NMR structure and the same holds for the relationships between the NMR and reference NMR structures relative to the crystal structure (Fig. 3 ▶ *b*).

Regarding the precision with which the experimental structures and the reference structures are defined, the present study coincides with previous observations on the treatment of the crystal structure with the NMR software (Jaudzems *et al.*, 2010 ▶). While the global r.m.s.d. for all heavy atoms in the crystal structure is nearly identical to the r.m.s.d. value obtained for the backbone heavy atoms, the r.m.s.d. values calculated for the corresponding selections of atoms in the reference crystal structure give values that differ by about twofold, which is similar to the corresponding ratio of the r.m.s.d. values for the NMR structure and the reference NMR structure (Fig. 3 ▶ *b*). In addition, we have the new observation that the all-heavy-atom r.m.s.d. between the two molecules in the crystal asymmetric unit is more than twofold greater than the corresponding backbone r.m.s.d.s. In this regard, the ‘bundle’ consisting of the two independent molecules in the crystal structure shows a similar behavior to the bundle of conformers that represent the NMR structure in solution (Fig. 3 ▶).

### 3.2. Precision along the amino-acid sequence in the NMR and crystal structures of TM1112   

**Figure 5:**
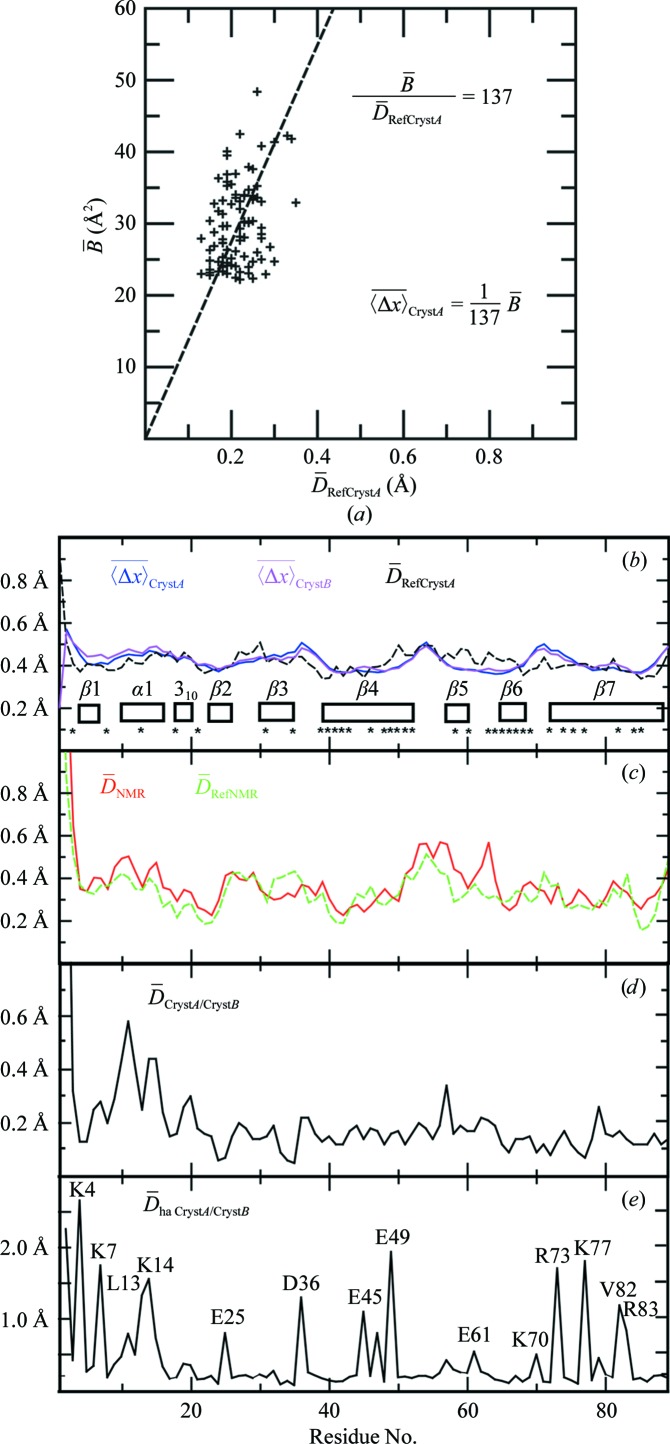
Per-residue 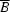 values for the backbone heavy atoms in Cryst*A* and Cryst*B* of TM1112, per-residue global backbone and all-heavy-atom displacements between Cryst*A* and Cryst*B* and mean values of the per-residue pairwise backbone displacements among the bundles of 20 conformers representing the NMR structure and the reference NMR and crystal structures. (*a*) Linear least-squares fit of the 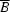 values for the Cryst*A* *versus* the corresponding displacements in the reference crystal structure, 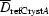. The resulting representation of the 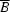 values by 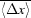 is used for comparisons with the 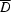 values for the other structures (Jaudzems *et al.*, 2010 ▶). (*b*)–(*d*) Plots of per-residue polypeptide backbone displacements *versus* the sequence. (*b*) Cryst*A* and Cryst*B* and reference crystal structure *A*. For crystal structure *B*, the same relation between 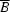 and 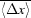 was used as for Cryst*A*. The locations of the regular secondary structures are indicated and asterisks identify the residues with solvent accessibility below 15% in the NMR structure. (*c*) NMR structure and reference NMR structure. (*d*) Backbone displacements between Cryst*A* and Cryst*B* in the crystal asymmetric unit. (*e*) All-heavy-atom displacements, 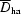, between Cryst*A* and Cryst*B*. Residues with large 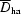 values are identified.

For comparisons at a resolution of individual amino-acid residues, we used the per-residue displacement, 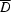, for the NMR structure and the two reference structures and an empirical determination of 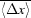 values by a linear fit of the 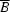 values for Cryst*A* to the 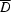 values of the reference crystal structure *A* (Fig. 5 ▶; Jaudzems *et al.*, 2010 ▶). The 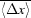 values for Cryst*A* and Cryst*B* vary in a narrow range of about ±0.05 Å along the amino-acid sequence and the same holds for the 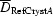 values (Fig. 5 ▶ *b*). The profile of per-residue displacements *versus* amino-acid sequence for the NMR structure shows larger variations than the crystal structure and is closely similar to that of the reference NMR structure. With the exception of helix α1, the regular secondary structures show lower 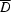 values than the intervening linker peptides (Fig. 5 ▶ *c*). The N-terminus of helix α1 (Thr9–Trp21) is involved in extensive crystal packing with the neighboring crystallographically related molecule, which could explain the shift *versus* the NMR structure. Overall, there is thus no indication in the data of Figs. 5 ▶(*b*) and 5 ▶(*c*) of any polypeptide segments with out­standing local structural differences, except possibly the apparent lower precision of the α1 helix in the NMR structure.

**Figure 6:**
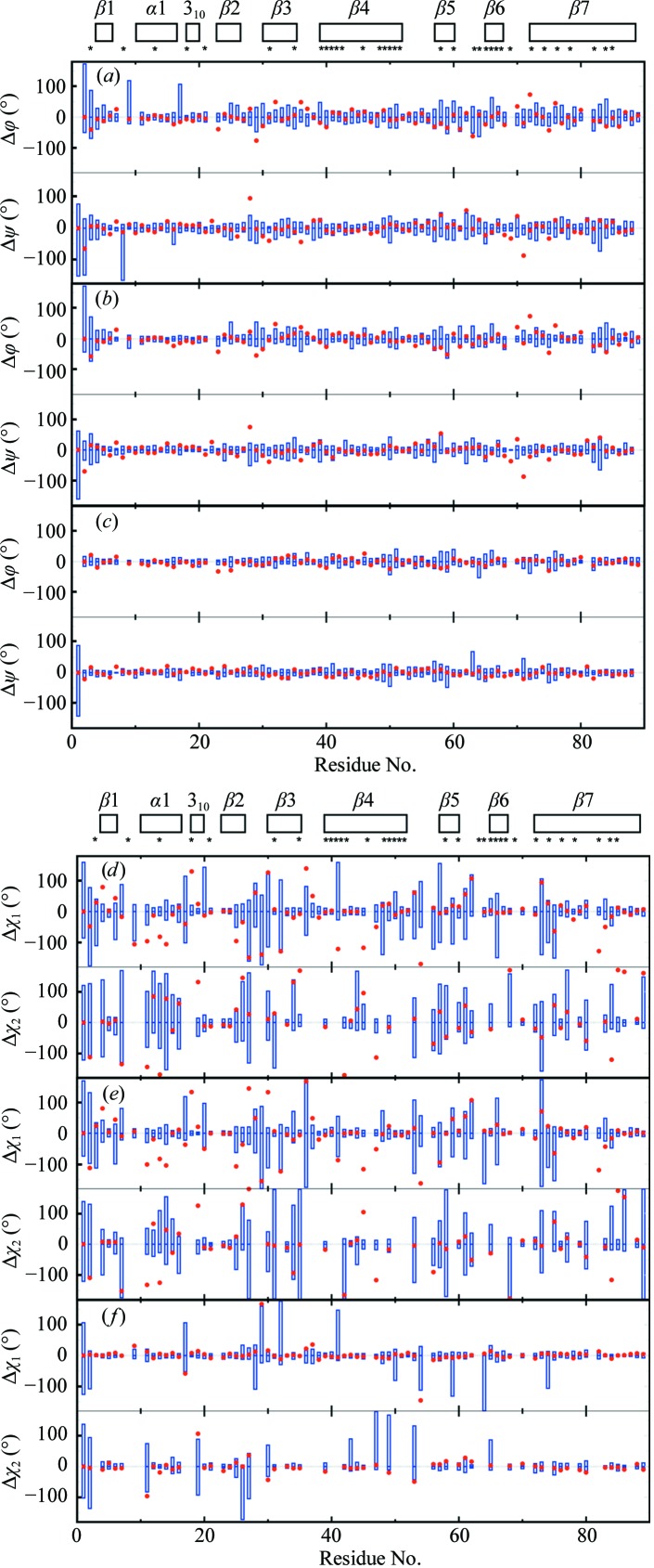
Variation of the backbone dihedral angles and side-chain torsion angles in the bundles of 20 energy-refined conformers representing the NMR structure and the reference structures (Fig. 3 ▶) of TM1112 and comparisons with Cryst*A*. The spread of the values for the backbone dihedral angles ϕ and ψ among the 20 conformers representing the NMR structure (*a*), the reference NMR structure (*b*) and the reference crystal structure *A* (*c*) (Fig. 3 ▶) is represented by blue vertical bars; the red dots indicate the deviations of the crystal structure values from the corresponding mean values for the bundles of 20 conformers, which are at 0°. (*d*)–(*f*) The same presentation as in (*a*) to (*c*) for the side-chain torsion angles χ_1_ and χ_2_ in the NMR structure (*d*), the reference NMR structure (*e*) and the reference crystal structure *A* (*f*). The locations of the regular secondary structures are indicated and asterisks identify the residues with solvent accessibility below 15% in the NMR structure.

**Figure 7:**
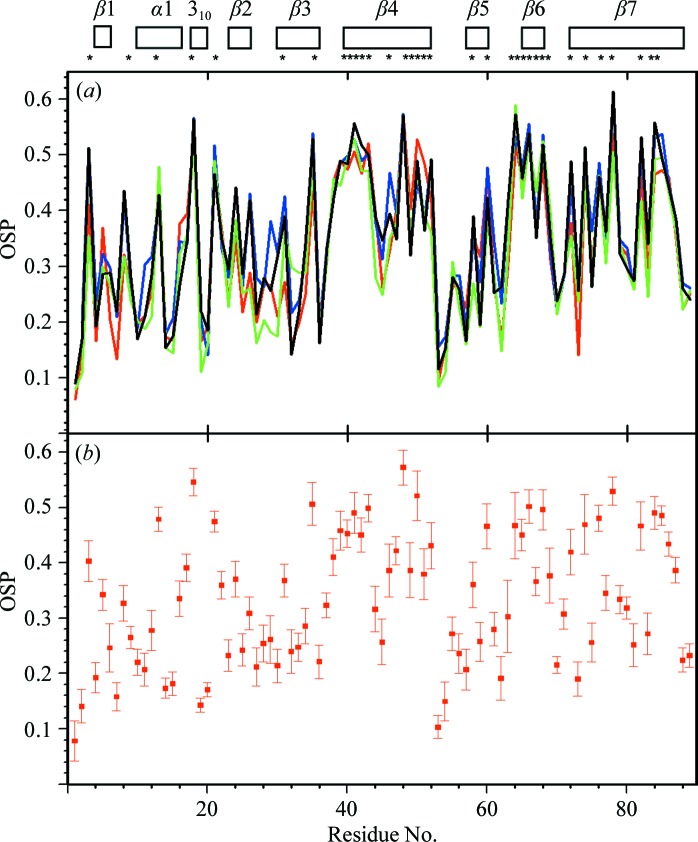
Occluded surface packing along the polypeptide chain of TM1112. (*a*) Plots *versus* the amino-acid sequence of the per-residue occluded surface packing (Pattabiraman *et al.*, 1995 ▶) for the NMR structure (red), Cryst*A* (blue), the reference NMR structure (green) and the reference crystal structure *A* (black). For the NMR structure and the two reference structures, the OSP values for the conformer closest to the mean atom coordinates are shown. At the top, the locations of the regular secondary structures are indicated; asterisks identify residues with solvent accessibility below 15% in the NMR structure. (*b*) Plot *versus* the amino-acid sequence of the mean per-residue OSP values in the bundle of NMR conformers and the standard deviations among the 20 NMR conformers.

The close fit between the NMR and crystal structures of TM1112 manifested by the global r.m.s.d. values (Fig. 3 ▶) can be rationalized by comparison of their torsion angles (Fig. 6 ▶). The backbone dihedral angles in the NMR structure are defined with high precision, with only five residues, Met1, Glu2, Pro8, Thr9 and Ser17, showing a spread of the ϕ and/or ψ values greater than ±100° among the 20 NMR conformers, where the crystal structure ϕ and ψ dihedral angles are within the range covered by the 20 NMR conformers. Overall, 85% of the ϕ and ψ dihedral angle values in the crystal structure lie within the ranges covered by the 20 NMR conformers. Deviations by more than 15° from the range covered by the NMR conformers are found only for 14 of the 89 residues, all of which are located in solvent-exposed loop regions of the protein (Fig. 6 ▶ *a*). The corresponding data for the reference NMR structure (Fig. 6 ▶ *b*) show qualitatively similar features, as seen in Fig. 6 ▶(*a*), and the reference crystal structure shows a very close coincidence with the crystal structure for the entire polypeptide chain; a spread greater than 50° in the reference crystal structure was observed only for the ψ values of residues 1 and 63 (Fig. 6 ▶ *c*). The χ_1_ side-chain torsion angles in the NMR structure show a large spread among the 20 conformers for 29 of the 89 residues and 34 residues show a large spread for χ_2_ (Fig. 6 ▶ *d*), but only 15 χ_1_ and 13 χ_2_ values of the crystal structure (only molecule *A* is shown) do not fall within the range covered by the 20 NMR conformers. The data of Fig. 6 ▶(*d*) are faithfully reproduced by the comparison of the reference NMR structure with the crystal structure (Fig. 6 ▶ *e*), with an apparent discrepancy seen only for the turn linking strands β2 and β3. The reference crystal structure shows a very close fit to the crystal structure (Fig. 6 ▶ *f*), despite large spreads of χ_1_ or χ_2_ values for residues devoid of non-labile H atoms in the peripheral atom groups (Jaudzems *et al.*, 2010 ▶). The profiles of the plots of the occluded surface packing (OSP; Pattabiraman *et al.*, 1995 ▶; Fleming & Richards, 2000 ▶) show similar patterns for the four experimental and reference structures, except for a strictly localized difference near the turn between β2 and β3 (residues 24–28; Fig. 7 ▶ *a*). OSP values of ≥0.4 are seen exclusively for residues with low solvent accessibility. Fig. 7 ▶(*b*) shows that the standard deviations for the bundle of 20 experimental NMR conformers are small when compared with the OSP variations along the sequence, which documents not only that the comparisons in Fig. 7 ▶(*a*) are meaningful, but that the variations of χ_1_ or χ_2_ in the bundle of NMR conformers (Fig. 6 ▶ *d*) are confined to ranges that are compatible with high packing density.

In the context of the present structure comparisons, the displacements between the two crystal structure molecules *A* and *B*, 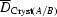 (Figs. 5 ▶ *d* and 5 ▶ *e*), are of special interest since they indicate that the ‘bundle’ of ‘conformers’ *A* and *B* in the crystal mimics the bundle of 20 NMR conformers in solution. Thus, the 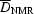 values in helix α1 are paralleled by high values of 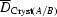 and even more pronounced correlations are seen for the all-heavy-atom per-residue displacements 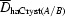 (Fig. 5 ▶ *e*). With the sole exceptions of Leu13 and Val82, large values are observed only for charged solvent-accessible residues, which coincides with the low precision of these side chains in the NMR structure. Likewise, high 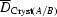 and 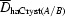 values do not correlate with the values for the corresponding 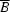 values in the individual molecules in the crystal structure (Fig. 5 ▶ *b*) and reflect actual differences of surface residues in Cryst*A* and Cryst*B*.

### 3.3. Comparison of the TM1112 NMR structures solved by the JCSG and the NESG   

We used the comparison of the NMR structures of TM1112 solved independently by the JCSG and the NESG as an additional criterion to evaluate the quality of the structure obtained with the new JCSG protocol, which uses *UNIO* (Herrmann *et al.*, unpublished work) for extensive automation. It is of special interest that the NESG NMR structure was used for molecular replacement to solve the JCSG crystal structure, so that comparison of the three structures might further provide insight into any possible bias in the techniques used.

**Figure 8:**
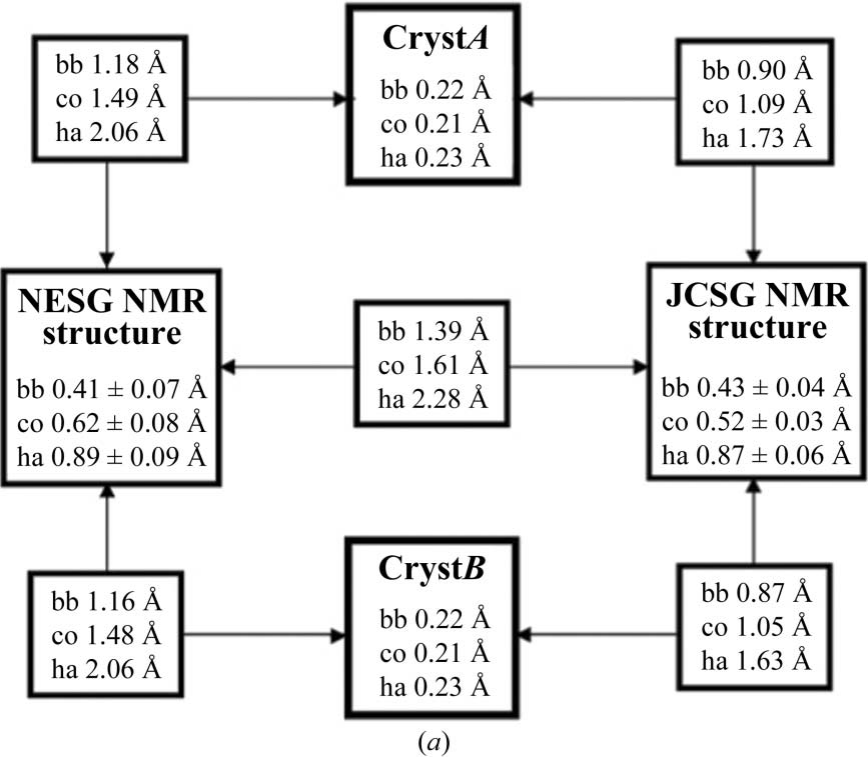

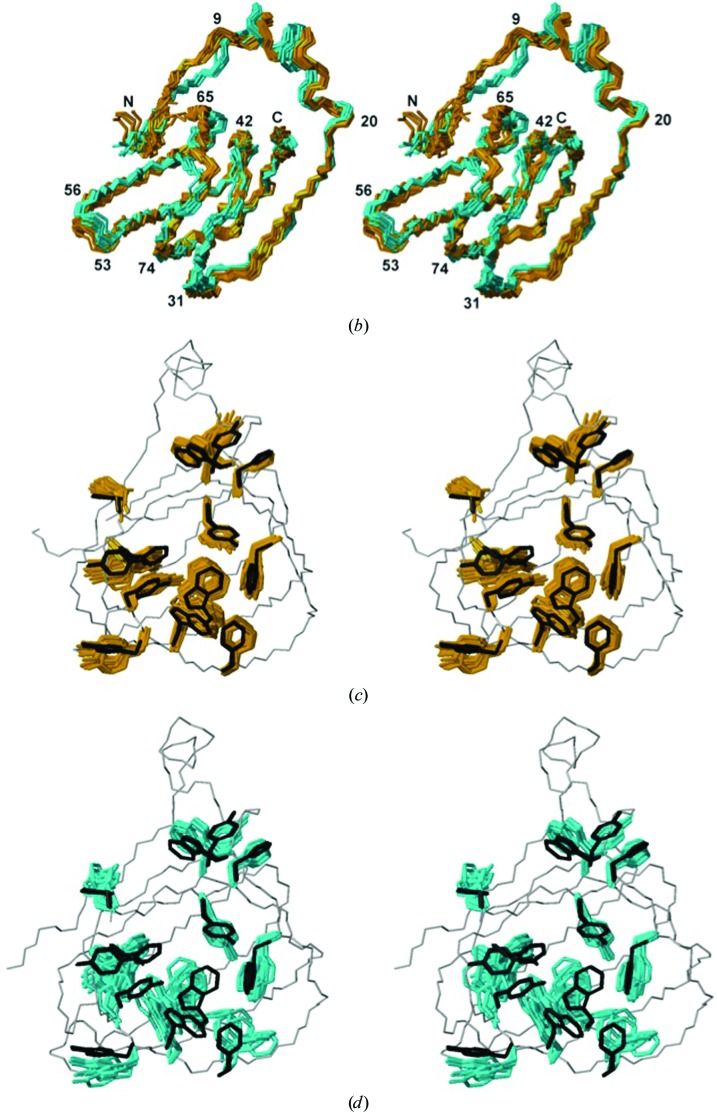
Comparison of the JCSG NMR structure and the NESG NMR structure of TM1112 with the crystal structure. (*a*) Global r.m.s.d. values describing the precision of the structure determinations of TM1112 by NMR and X-ray crystallography and pairwise comparisons of the four structures, as in Fig. 3 ▶. (*b*) Stereoview of a superposition for best fit of the polypeptide backbone heavy atoms of residues 2–89 of the NMR structures solved by the JCSG (brown; PDB code 2k9z) and the NESG (cyan; PDB code 1lkn). When generating this picture, we first computed the mean atom coordinates of the 20 JCSG NMR conformers (Fig. 1 ▶ *b*) and identified the conformer closest to the mean. Each of the other 19 JCSG NMR conformers and the ten NESG NMR conformers were then superimposed for best fit of the backbone heavy atoms with this conformer. (*c*) Stereoview of the aromatic side chains in the bundle of 20 conformers representing the JCSG NMR structure (brown) superimposed with Cryst*A* and Cryst*B* (black). (*d*) The same presentation as in (*c*) for the NESG bundle of ten NMR conformers, with the aromatics in cyan. For ease of orientation in (*c*) and (*d*), the backbone of the best NMR conformer in each bundle is indicated with a thin line. When generating the drawings (*c*) and (*d*), Cryst*B* and the 20 JCSG NMR conformers or the ten NESG NMR conformers, respectively, were superimposed for best fit of the polypeptide-backbone heavy atoms with Cryst*A*.

Fig. 8 ▶(*a*) shows that the two NMR structures have been determined with nearly identical precision and that the r.m.s.d.s for the structure comparisons exceed those of the two separate bundles by about threefold. Pairwise comparisons of the NMR structures with Cryst*A* and Cryst*B* show the closest fits between the JCSG NMR structure and the crystal structures. It is remarkable that although the NESG structure was used to determine the crystal structure by molecular replacement, the r.m.s.d.s with the crystal structure are significantly larger than those for the JCSG NMR structure and the crystal structure; it is notable that when side-chain atoms are included in the comparison the r.m.s.d. values increase significantly (bb *versus* co or ha in Fig. 8 ▶ *a*). The local origins of the contributions to the global r.m.s.d.s of Fig. 8 ▶(*a*) are visualized by the structure superpositions in Figs. 8 ▶(*b*)–8 ▶(*d*), where the lower definition of the core residues in the NESG NMR structure is clearly illustrated by Fig. 8 ▶(*d*). We conclude that the new JCSG NMR structure-determination protocol with automation through the use of the *UNIO* interface (Herrmann *et al.*, unpublished work) yielded a structure that compares favorably with a structure obtained using a conventional interactive method. Since the JCSG NMR structure exhibits a closer fit with the crystal structure coordinates obtained by molecular replacement with the NESG NMR structure, further evidence is provided that the crystal structure determination was not biased by the molecular-replacement model used.

### 3.4. Comparison of the NMR and crystal structures of TM1367   

The NMR structure was solved at 298 K in 25 m*M* sodium phosphate buffer pH 6.0, 50 m*M* sodium chloride, 5 m*M* DTT and 0.03%(*w*/*v*) sodium azide. The crystal structure was determined to 1.90 Å resolution at 100 K using a crystal obtained at 277 K from 100 m*M* phosphate–citrate buffer pH 4.2, 200 m*M* sodium chloride and 50%(*w*/*v*) PEG 200 (Jin* et al.*, 2006 ▶). The NMR structure of TM1367 comprises 11 β-strands consisting of residues 2–7, 11–16, 32–34, 37–38, 42–44, 57–58, 65–69, 74–78, 95–96, 99–103, 117–120 and one α-helix and one 3_10_-helix consisting of residues 21–29 and 105–110, respectively, as illustrated in Fig. 2 ▶(*c*). The structure contains a nine-stranded antiparallel β-barrel composed of strands β4–β5–β8–β7–β10–β2–β1–β11–β3 that connect to a two-stranded antiparallel sheet composed of β6 and β9 at β7 (Fig. 2 ▶ *a*). The two helices cover the open ends of the β-barrel. Since the β-barrel is strongly twisted, the axes of the two helices are approximately perpendicular to each other. Statistics for the NMR structure determination of TM1367 are given in Table 2 ▶ and those for the crystal structure have been presented elsewhere (Jin *et al.*, 2006 ▶). The comparison of the three molecules in the crystal structure of TM1367 with the bundles of 20 conformers representing the NMR structure and the reference structures yielded very similar results as observed for TM1112 (Figs. 2 ▶, 4 ▶, 9 ▶, 10 ▶ and 11 ▶). It is worth noticing that the 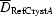 value for residue Gly40 is not reproduced by the 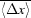 values in the crystal structure. The lower precision for Gly40 in the reference crystal structure is clearly related to the use of the NMR software with a low number of constraints for Gly, as indicated by the coincidence with the NMR structure and the NMR reference structure. Overall, this more complex structure was determined with similar precision and comparable coincidence with the crystal structure. While the complete data are presented in the aforementioned figures, we limit the following discussion to selected features that support key results from the investigation of TM1112 as well as from previous studies (Jaudzems *et al.*, 2010 ▶).

In terms of the global r.m.s.d. values (Fig. 4 ▶), the bundle of three crystal ‘conformers’ mimics the behavior of the bundle of 20 NMR conformers in that the all-heavy-atom r.m.s.d. values are about twofold larger than the corresponding backbone r.m.s.d.s and the same is observed for the reference crystal structure. The somewhat smaller heavy-atom r.m.s.d.s with respect to TM1112 are a consequence of the fact that some side chains of solvent-exposed residues in Cryst*B* and Cryst*C* have been truncated owing to a lack of interpretable electron density (Jin *et al.*, 2006 ▶).

**Figure 9:**
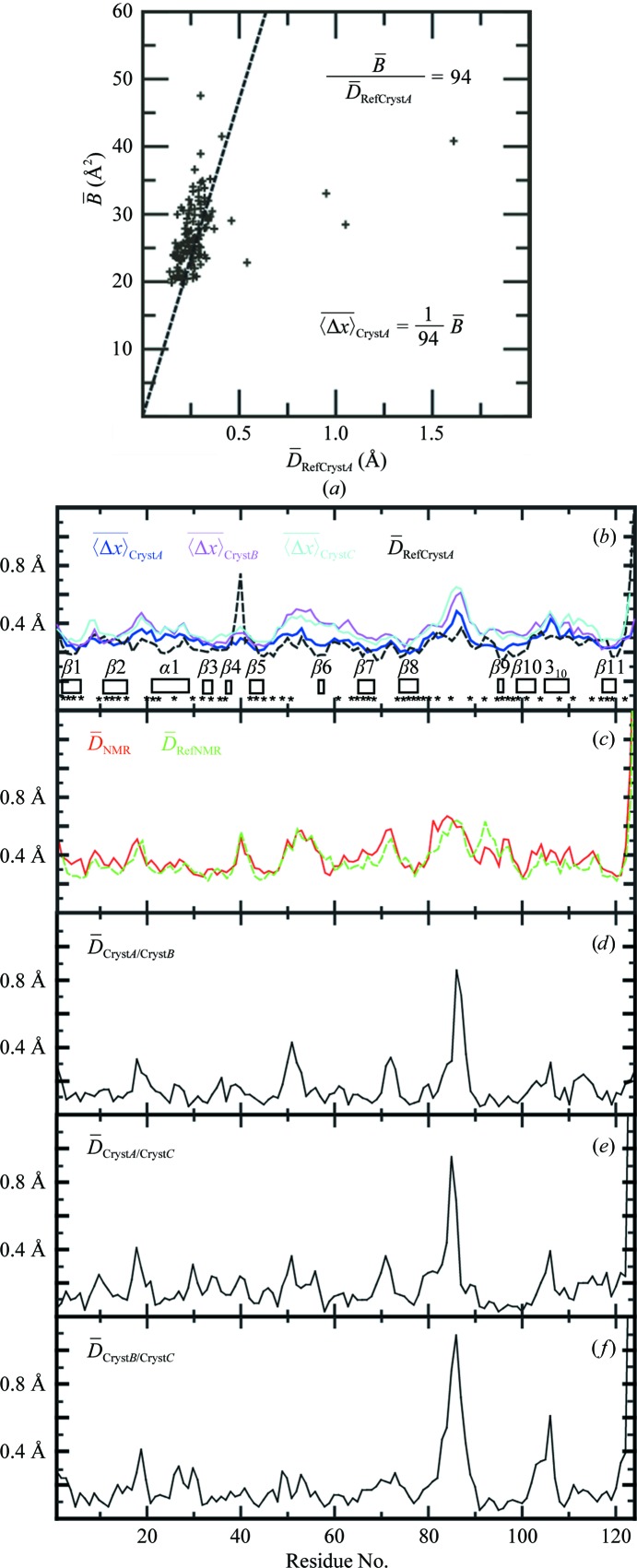
Per-residue 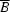 values of the backbone heavy atoms in Cryst*A*, Cryst*B* and Cryst*C* of TM1367, per-residue pairwise global displacements between the three structures in the asymmetric unit of the crystal and mean values of the per-residue pairwise global displacements among the bundles of 20 conformers representing the NMR and reference structures. The same presentation is used as in Fig. 5 ▶, except that no 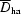 data are given (see text).

The residue-by-residue data on the precision of the individual structure determinations and the structure comparisons (Figs. 9 ▶, 10 ▶ and 11 ▶) document a close coincidence of the different structures, similar to TM1112. Furthermore, as in TM1112 the regions with the largest pairwise displacements among Cryst*A*, Cryst*B* and Cryst*C* (Figs. 9 ▶ *d*, 9 ▶ *e* and 9 ▶ *f*) correlate with similar increased variation among the NMR conformers (Fig. 9 ▶ *c*); notwithstanding, the structure variations among the molecules in the crystal are more pronounced and these increased displacements are only seen for polypeptide segments with nonregular secondary structure. A particular case is the prominent peaks in Figs. 9 ▶(*d*), 9 ▶(*e*) and 9 ▶(*f*), which seem to correspond to regions with significantly different environments in the a.s.u., such as the loop 85–89, which is involved in a crystal contact in Cryst*A*, is in a solvent channel in Cryst*B*, and in Cryst*C* contacts the His tag of Cryst*A*. Another example is the large displacement observed for Glu106, which in Cryst*C* interacts with the side chain of Glu62 of a symmetry-related molecule *via* a water molecule. In contrast to TM1112, the residues with the largest variations among molecules Cryst*A*, Cryst*B* and Cryst*C* also have the largest 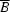 values (this analysis was not extended to the all-heavy-atom displacements because of the aforementioned high percentage of truncated side chains in Cryst*B* and Cryst*C*; PDB entry 1zx8).

## 4. Conclusions   

The present comparisons of the NMR and crystal structures of the proteins TM1112 and TM1367 provide additional support that the new JCSG NMR structure-determination protocol, which includes extensive automation through use of the *UNIO* software (Herrmann *et al.*, 2002*a* ▶,*b* ▶; Volk *et al.*, 2008 ▶; Fiorito *et al.*, 2008 ▶; Herrmann *et al.*, unpublished work), yields highly precise and accurate structures of small globular proteins which compare favorably with the results from more highly interactive and time-consuming conventional procedures. An interesting new insight has emerged from this analysis of the multiple molecules or ‘conformers’ in the crystal asymmetric unit, since these ‘bundles of conformers’ reproduced features that were observed in solution for the ensemble of NMR conformers both with regard to global r.m.s.d.s as well as to residue-by-residue com­parisons along the amino-acid sequence. However, the situation in the crystal is not strictly analogous to that in solution as the different molecules in the crystal asymmetric unit may have different chemical environments and, hence, adopt slightly different structures depending on the environment. Furthermore, at ultrahigh resolution, truly different conformers may be able to be discerned and interpreted for each of the molecules in the crystal structure either at the side-chain or at the backbone level. Nevertheless, at the presently achieved resolution of about 1.8 Å, superposition of the different molecules in the asymmetric unit emulates the conformational polymorphisms seen by NMR in solution, as has been implicated by a wide range of previous studies (*e.g.* Wilson & Brunger, 2000 ▶; DePristo *et al.*, 2004 ▶; Furnham *et al.*, 2006 ▶; Levin *et al.*, 2007 ▶; Kondrashov *et al.*, 2008 ▶). Furthermore, the recently introduced strategy of ‘reference structures’, which are obtained by treatment of the crystal structural data with the NMR software, validates and supports the information derived from the detailed structure comparisons in the crystal and in solution. In particular, the reference crystal structure clearly manifests the same features that are derived from comparison of the multiple independent structures in the crystal. Thus, this extensive analysis of the comparisons of the crystal and NMR structures suggests that either individual molecules or groups of two or several molecules in the asymmetric unit of the crystal can provide valuable indications about the conformational polymorphisms that are present in solution.

## Supplementary Material

PDB reference: TM1112, 2k9z

PDB reference: TM1367, 2ka0

## Figures and Tables

**Figure 10:**
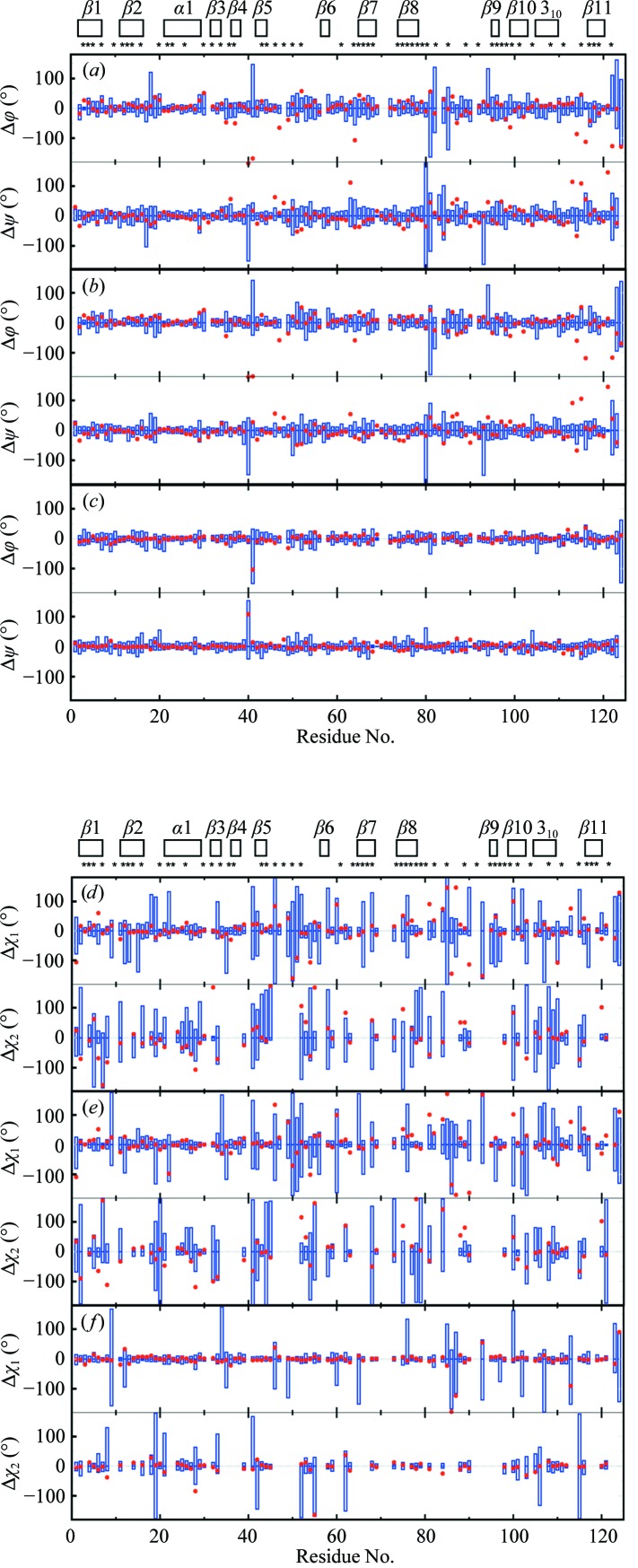
Variation of the backbone dihedral angles in the bundles of 20 energy-refined conformers representing the NMR structure and the reference structures (Fig. 4 ▶) of TM1367 and comparisons with Cryst*A*. The same presentation is used as in Fig. 6 ▶.

**Figure 11:**
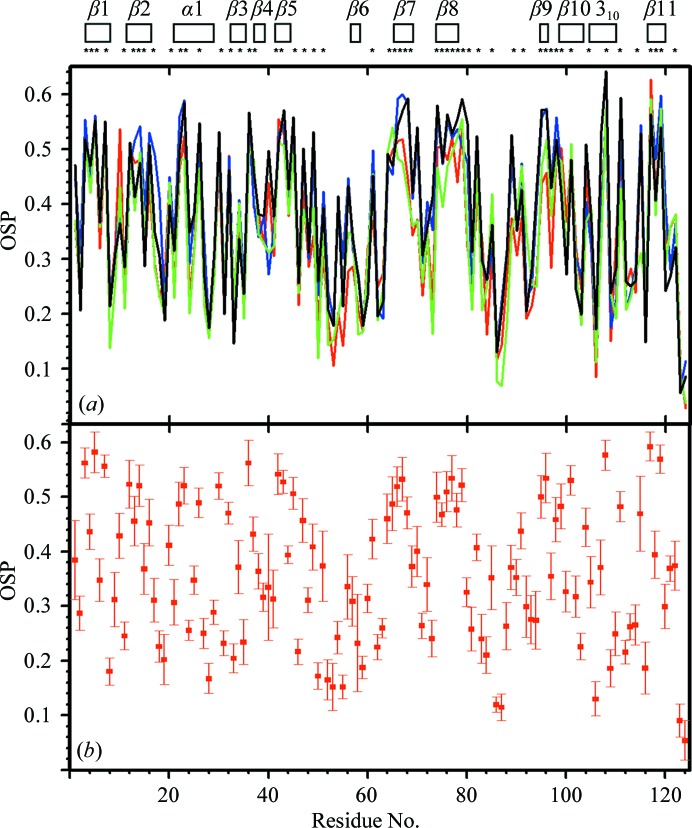
Occluded surface packing along the polypeptide chain of TM1367. The same presentation is used as in Fig. 7 ▶. The crystal structure is represented by Cryst*A* and RefCryst*A*.

**Table 1:** Determination of the NMR structure, a reference crystal structure and a reference NMR structure of the protein TM1112: input for the structure calculations and characterization of bundles of 20 energy-minimized *CYANA* conformers representing the structures Except for the top six entries, average values and standard deviations for the 20 energy-minimized conformers are given.

	NMR structure†	Reference crystal structure‡	Reference NMR structure§
NOE upper distance limits	2189	4125	3525
Intra-residual	514	937	1017
Short-range	590	943	944
Medium-range	329	592	410
Long-range	756	1653	1154
Dihedral angle constraints	406	353	351
Residual target-function value (^2^)	1.59 0.72	0.92 0.15	0.75 0.1
Residual NOE violations			
No. 0.1	12 6	3 2	1 1
Maximum ()	0.17 0.12	0.11 0.01	0.10 0.03
Residual dihedral angle violations			
No. 2.5	1 1	1 1	1 1
Maximum ()	5.9 4.5	2.35 1.15	2.32 0.5
AMBER energies (kcalmol^1^ ¶)			
Total	3593 239	3980 98	3733 89
van der Waals	300 97	379 11	346 10
Electrostatic	4046 119	4283 88	4075 88
R.m.s.d. from mean coordinates†† ()			
Backbone (289)	0.43 0.04	0.23 0.04	0.37 0.03
All heavy atoms (289)	0.87 0.06	0.57 0.05	0.85 0.06
Ramachandran plot statistics‡‡ (%)			
Most favored regions	83.3	89.7	86.3
Additional allowed regions	16.0	10.3	13.7
Generously allowed regions	0.6	0	0
Disallowed regions	0.1	0	0

† Structure calculated from the experimental NMR data. The top six entries represent the input generated in the final cycle of the *ATNOS*/*CANDID* and *CYANA* calculation.

‡ Structure calculated with *CYANA* from conformational constraints derived from the molecular model representing the crystal structure (Jaudzems *et al.*, 2010 ▶).

§ Structure calculated with *CYANA* from conformational constraints derived from the bundle of 20 molecular models representing the NMR structure (Jaudzems *et al.*, 2010 ▶).

¶ 1 kcal = 4.186kJ.

†† The numbers in parentheses indicate the residues for which the r.m.s.d. was calculated.

‡‡ As determined by *PROCHECK* (Laskowski *et al.*, 1993 ▶). The crystal structure (1o5u) deposited in the PDB has 96.8% of residues in favored regions, 3.2% additionally allowed, 0% generously allowed and 0% disallowed.

**Table 2:** Determination of the NMR structure, a reference crystal structure and a reference NMR structure of the protein TM1367: input for the structure calculations and characterization of bundles of 20 energy-minimized *CYANA* conformers representing the structures Except for the top six entries, average values and standard deviations for the 20 energy-minimized conformers are given.

	NMR structure†	Reference crystal structure‡	Reference NMR structure§
NOE upper distance limits	3028	5412	4730
Intra-residual	612	1107	1266
Short-range	779	1251	1299
Medium-range	443	825	682
Long-range	1194	2229	1483
Dihedral angle constraints	520	452	463
Residual target-function value (^2^)	2.50 0.62	1.21 0.27	1.40 0.15
Residual NOE violations			
No. 0.1	33 5	4 2	2 1
Maximum ()	0.27 0.26	0.11 0.02	0.10 0.01
Residual dihedral angle violations			
No. 2.5	1 1	1 1	1 1
Maximum ()	3.16 1.28	3.19 0.87	1.99 0.5
AMBER energies (kcalmol^1^ [Table-fn tfn10])			
Total	4711 186	5285 99	4886 104
van der Waals	396 28	531 17	454 15
Electrostatic	5313 132	5638 99	5314 100
R.m.s.d. from mean coordinates[Table-fn tfn11] ()			
Backbone (2123)	0.44 0.05	0.29 0.03	0.40 0.04
All heavy atoms (2123)	0.84 0.07	0.61 0.04	0.83 0.06
Ramachandran plot statistics[Table-fn tfn12] (%)			
Most favored regions	71.1	85.2	75.8
Additional allowed regions	27.5	14.8	22.8
Generously allowed regions	0.9	0	0.7
Disallowed regions	0.5	0	0.7

† Structure calculated from the experimental NMR data. The top six entries represent the input generated in the final cycle of the *ATNOS*/*CANDID* and *CYANA* calculation.

‡ Structure calculated with *CYANA* from conformational constraints derived from the molecular model representing the crystal structure (Jaudzems *et al.*, 2010 ▶).

§ Structure calculated with *CYANA* from conformational constraints derived from the bundle of 20 molecular models representing the NMR structure (Jaudzems *et al.*, 2010 ▶).

¶ 1 kcal = 4.186kJ.

†† The numbers in parentheses indicate the residues for which the r.m.s.d. was calculated.

‡‡ As determined by *PROCHECK* (Laskowski *et al.*, 1993 ▶). The crystal structure (1zx8) deposited in the PDB has values of 98.4% of residues in favored regions, 1.6% additionally allowed, 0% generously allowed and 0% disallowed.
